# Bioavailability of Oral Curcumin in Systematic Reviews: A Methodological Study

**DOI:** 10.3390/ph17020164

**Published:** 2024-01-28

**Authors:** Viljemka Bučević Popović, Esma Karahmet Farhat, Ines Banjari, Antonia Jeličić Kadić, Livia Puljak

**Affiliations:** 1 Department of Chemistry, Faculty of Science, University of Split, 21000 Split, Croatia; viljemka@pmfst.hr; 2 Department of Food and Nutrition Research, Faculty of Food Technology, University of Osijek, 31000 Osijek, Croatia; ekarahmet@ptfos.hr (E.K.F.); ines.banjari@ptfos.hr (I.B.); 3 Department of Pediatrics, University Hospital Split, 21000 Split, Croatia; jelicic.antonia@gmail.com; 4 Center for Evidence-Based Medicine and Health Care, Catholic University of Croatia, 10000 Zagreb, Croatia

**Keywords:** bioavailability, curcumin, nutraceutical, turmeric, systematic review

## Abstract

Curcumin is a natural compound found in turmeric that exhibits diverse biological activities. However, its poor bioavailability limits its therapeutic application, which has led to the development of various bioavailability-improved formulations. In this methodological study, we analyzed whether systematic reviews on curcumin considered the bioavailability of systemic oral curcumin formulations when synthesizing evidence from human clinical trials. A total of 171 systematic reviews published between 2003 and 2022 were included in the study. From the included studies, we extracted data on study characteristics; type of curcumin; methods; and reporting regarding bioavailability, funding, and conflict of interest. Our results show that systematic reviews rarely consider the variable bioavailability of tested curcumin formulations. Relevant statistical subgroup and/or sensitivity analyses were reported in the methods and results of only 3.5% and 6.4% of reviews, respectively. However, more reviews mentioned bioavailability in their discussion (57%) or conclusion (13%). The detailed analysis of the included systematic reviews suggests that there is broad recognition of product bioavailability as a crucial factor affecting the health effects of curcumin, which is not accompanied by adequate evidence synthesis. Therefore, the results of most systematic reviews on orally administered curcumin should be taken with caution.

## 1. Introduction

Turmeric (*Curcuma longa* L.) is a widely used spice in Asian cuisine, as a food coloring ingredient, and in traditional Indian (Ayurvedic) and Chinese medicine. Its health benefits are mainly attributed to curcumin, a major polyphenol compound that naturally occurs in the root of turmeric and other Curcuma species [1,2]. Chemically, curcumin is described as 1,7-bis(4-hydroxy-3-methoxyphenyl)-1,6-heptadiene-3,5-dione, which has a lipophilic body and hydrophilic groups. The molecule exhibits keto-enol tautomerism, which is pH-dependent and linked to its biological properties [3].

Over the past five decades, curcumin has undergone extensive research, which showed it is a potent pharmacological molecule with a broad spectrum of biological activities proven in vitro [4,5]. Among its many positive attributes, curcumin displays antioxidant, antimicrobial, anti-inflammatory, and anticancer properties. It also exhibits beneficial effects in conditions like diabetes, depression, immune deficiencies, and various other human diseases [1,6].

However, despite its promising biological effect, the use of curcumin as a therapeutic agent has been significantly limited by its poor bioavailability [7]. Bioavailability implies the fraction of curcumin from the total intake that passes into the bloodstream. It has been highlighted in many studies that low curcumin bioavailability following oral systemic intake is a critical issue for achieving therapeutic concentration in the blood [8,9,10,11,12]. Numerous factors contribute to the unfavorable pharmacokinetics of curcumin: very low water solubility (approximately 11 ng/mL) [13,14], poor absorption in the gut, fast metabolism as well as low distribution, and fast excretion [15,16]. Curcumin is metabolized mainly by the enzymes found in the intestine, liver, and kidneys. Phase 1 metabolism comprises the reduction of curcumin double bonds by NADPH-dependent reductases, producing dihydro-, tetrahydro-, hexahydro-, and octahydrocurcumin. Curcumin and its phase 1 metabolites are further metabolized by phase 2 conjugation reactions to yield highly soluble glucuronide and sulfate conjugates that are rapidly eliminated by feces and urine. The gut microbiota has also been shown to participate in curcumin metabolism through a metabolic pathway involving microbial reductase [2,6]. Most of the ingested curcumin is eliminated from the human body through the gut, while plasma concentrations remain low even when high oral doses are administered [9,17]. The study by Dhillon et al. reported curcumin plasma concentrations of only 21–41 ng/mL in advanced pancreatic cancer patients receiving a daily dose of 8 g of curcumin. More importantly, curcumin was mainly present in the form of glucuronide and sulfate conjugates that do not display the same bioactivities attributed to ‘free’ curcumin [18].

Possible solutions for increasing bioavailability are offered through the reduction in metabolism through the liver by additives such as piperine (inhibitor of curcumin glucuronidation), curcumin derivatization, a formulation of complexes with cyclodextrins, liposomes, micelles, emulsions, solid dispersions, and various nanoparticles. The innovative formulations have demonstrated an increase in curcumin bioavailability, ranging from 6.9- to 185-fold [7]. A comprehensive overview of the strategies used to improve the pharmacokinetics and bioavailability of oral curcumin formulations has been provided in several recent reviews [2,3,11,12,17,19,20,21].

We have previously shown that randomized controlled trials (RCTs) about oral curcumin as an intervention frequently use methods for enhancing curcumin bioavailability, but they use many different methods. The effect of different methods was seldom compared head-to-head in those trials. Thus, it is difficult to know which of those methods is optimal for enhancing curcumin bioavailability [8]. It is not known how the problem of curcumin bioavailability is addressed in systematic reviews.

A systematic review is an evidence synthesis aiming to answer a specific research question using a specific methodology [22,23]. A meta-analysis presents the quantitative synthesis of different studies and results and may be used in a systematic review [24]. If systematic reviews about the effects of oral curcumin do not account for the different bioavailability methods used in their qualitative and quantitative synthesis, this would adversely impact the review’s conclusions.

This study aimed to analyze whether systematic reviews of curcumin considered the bioavailability of systemic oral curcumin when synthesizing evidence from included studies; whether different bioavailability enhancement methods were considered in the statistical analysis of the results, namely for subgroup and sensitivity analyses; and whether they discussed their results in terms of curcumin bioavailability.

## 2. Results

**Figure 1:**
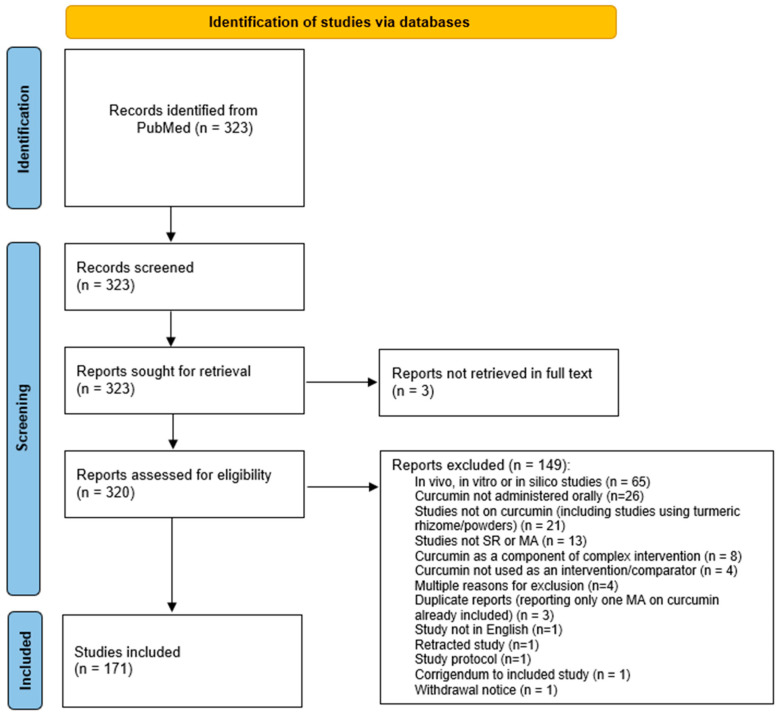
Flow diagram according to PRISMA (Preferred Reporting Items for Systematic Reviews and Meta-Analysis) guidelines.

We retrieved a total of 323 records via a search. We excluded 149 records that were not eligible due to reasons reported in Supplementary File S1. We included 171 systematic reviews in the analysis. The list of included studies accompanied with full bibliographic references and all extracted data used for our analysis is reported in Supplementary File S2. The flow diagram of study selection is shown in Figure 1.

### 2.1. Characteristics of Included Systematic Reviews

**Figure 2:**
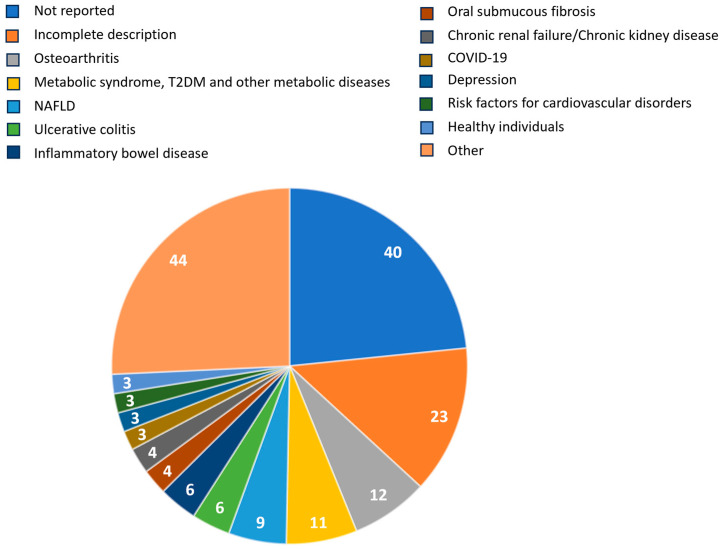
Type of participants included in oral curcumin systematic reviews (N = 171) as reported in the methods (acronyms: NAFLD = non-alcoholic fatty liver disease; T2DM = type 2 diabetes mellitus).

The included systematic reviews were published between 2003 and 2022. The majority included only RCTs (Table 1). The median number of included studies was six, and the median number of included participants was 380. In the methods, the included population was not reported in 40 (24%) studies (Figure 2). In an additional 23 (13%) studies, reporting on the study population was incomplete as no clear inclusion criteria were provided, but only general remarks on the study population (e.g., ‘adults’, ‘adult men and women’). The most common types of participants reported in the methods were those with osteoarthritis, metabolic diseases, non-alcoholic fatty liver disease, ulcerative colitis, and inflammatory bowel disease (Figure 2).

Most frequently, studies reported in the methods that the intervention analyzed was ‘curcumin’ (33%). There were 21% of systematic reviews that did not specify in the methods what kind of intervention they used (Table 1). Most reviews (70%) contained a statement about review funding. Among those that did report a source of funding, most were funded by non-commercial sources. In 4.1% of reviews, at least one author was funded/supported by a sponsor of the investigated product (Table 1).

### 2.2. Mentions of Bioavailability

**Figure 3:**
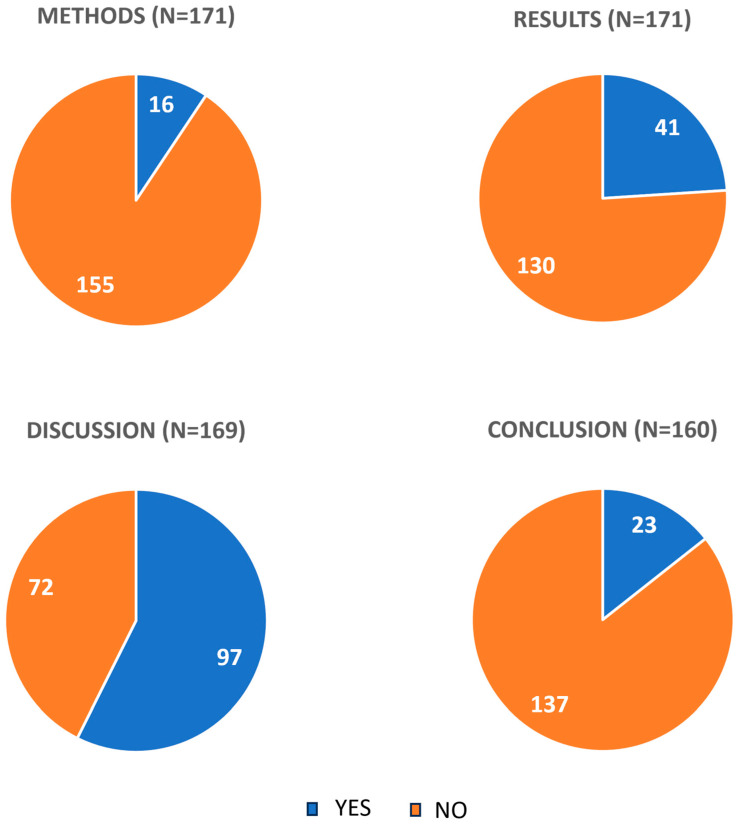
The number of studies mentioning bioavailability (Yes = bioavailability mentioned; No = bioavailability not mentioned) in specified sections. On some occasions, studies did not include a discussion and/or conclusion, and the number of studies analyzed (N) was <171.

There were 16 (9.3%) reviews that mentioned bioavailability in the review methods (Figure 3). Most of those reviews mentioned bioavailability in terms of inclusion criteria (i.e., a description of the type of curcumin product that was considered eligible; N = 6; 3.5%) and statistics (N = 6; 3.5%) (Table 2). Statistical analyses mentioned in the methods regarding bioavailability enhancement methods of reviews referred to subgroup analysis (N = 5) and sensitivity analysis (N = 1). For subgroup analysis, studies were mostly subdivided into two categories based on the type of curcumin product used, such as bioavailability-enhanced vs. non-bioavailability-enhanced or high vs. low bioavailability [25,26,27]. In one of the reviews, they were subdivided into three categories: low bioavailability, high bioavailability, and nanocurcumin [28].

Bioavailability was mentioned in the review results in 41 (24%) reviews (Figure 3). Those reviews mentioned bioavailability mostly to describe interventions that were used in the included studies (N = 19; 11%) (Table 2). In 10 (5.8%) reviews, the results of a subgroup analysis for bioavailability were reported [25,26,27,28,29,30,31,32,33,34]. In the systematic review by Al-Karawi et al., two studies using BCM-95 (bioavailability improved formulation of curcumin and turmeric essential oil) were erroneously classified to the subgroup ‘curcumin without enhancers’ [25]. The study by Bannuru et al. included an intervention with NR-INF-02, a curcumin-free turmeric polysaccharide formulation in the ‘enhanced-bioavailability’ curcumin product subgroup [29]. Another study assessed not only curcumin but all types of turmeric extracts in terms of their bioavailability, both curcumin- and polysaccharide-enriched [28]. The results of the subgroup analysis for the remaining seven studies are summarized in Table 3. In most of the studies, the favorable effects on intervention outcomes were smaller or completely absent in the non-bioavailability-improved subgroup. One study (0.6%) reported the results of a sensitivity analysis for bioavailability [35]. Sensitivity analysis was conducted by excluding studies using high-bioavailability curcumin nanomicelles from the set of studies using other curcumin forms, but the overall results did not change. There were seven additional (4.1%) reviews that provided results for studies using bioavailability enhancement and compared them narratively with other products. Few studies mentioned bioavailability in terms of reporting plasma levels of curcumin for different products, methodological assessment, the lack of studies that have evaluated bioavailability in different curcumin formulations, the stratification of results, or explaining causes of the low oral availability of curcumin (Table 2).

The bioavailability of curcumin was mentioned in 97 (57%) of reviews in the discussion (Figure 3). Most of the studies mentioned general comments about poor bioavailability of curcumin in the discussion (40%) (Table 2). In 13% of studies, bioavailability was mentioned in descriptions of included interventions, and 11% commented on the results of the studies in terms of products with enhanced availability. Recommendations for future trials in terms of including studies of products with enhanced bioavailability were provided in 8.8% of reviews.

The bioavailability of curcumin was mentioned In the conclusion in 23 (13%) reviews (Figure 3). In most of those reviews, authors summarized the results they obtained regarding the interventions in terms of their bioavailability and provided recommendations for future studies regarding curcumin bioavailability (Table 2).

## 3. Discussion

Oral bioavailability is an essential component of the bio-efficiency of bioactive natural compounds. Curcumin has low aqueous solubility, and it is rapidly metabolized in the gastrointestinal system, resulting in low oral bioavailability that limits its medicinal potential. This study analyzed systematic reviews on orally administered curcumin to assess if the bioavailability of specific curcumin products was considered when synthesizing evidence from clinical trials. We found that 9.3% of analyzed systematic reviews mentioned bioavailability in the methods, 24% in the results, 57% in the discussion, and 13% in the conclusion. Statistics that took into account interventions depending on bioavailability, such as subgroup analyses and sensitivity analyses, were reported in only 3.5% of reviews in methods and 6.4% in results. These findings indicate that systematic reviews insufficiently consider the problem of bioavailability when synthesizing evidence from human curcumin trials, and thus, their results should be taken with caution.

It is reasonable to expect that different formulations of curcumin and different methods used to improve its oral bioavailability will impact human clinical trials. However, our data on this are limited due to the paucity of data in the literature about comparisons of different bioavailability methods in clinical trials. This was shown by our recent study, published in 2022, which analyzed whether bioavailability was mentioned and used in RCTs investigating systematic oral curcumin. We analyzed 165 RTs published until September 2020 and showed that 107 (64%) of the trials reported that they used some method for enhancing the oral bioavailability of curcuminoids [8]. However, this study [8] indicated that 25 different interventions for enhancing curcumin bioavailability were used in the 107 trials. Few trials have directly compared the effects of different methods to enhance curcumin bioavailability. We concluded that curcumin bioavailability was insufficiently considered in a third of the analyzed trials. Furthermore, we concluded that new trials are warranted to study different curcumin-containing products’ comparative bioavailability and efficacy [8]. Our results are in line with those of Panknin et al. [36]. In their scoping review from 2023, they analyzed 389 studies that used oral curcumin in different disease patients. The authors indicated that curcumin may affect disease conditions, with the strongest evidence obtained for diseases driven by inflammation. However, they also highlighted that the studies used various bioavailability-enhanced products, making a comparison of clinical trial effects difficult [36].

In this study, we included systematic reviews as units of analysis. We would expect that systematic reviews should take into account potential differences between interventions used in the included studies. As we have shown previously, primary studies can include highly heterogeneous curcumin products [8]. Consequently, systematic reviews should account for potentially different interventions by incorporating adequate subgroup analyses in their protocols. Studies using different curcumin interventions with different bioavailability methods should not be considered as a single intervention. On the contrary, systematic reviews should compare the effects of different curcumin interventions, depending on which bioavailability enhancement methods were used in a product, if any.

Our findings were very disappointing in that respect, as only 24% of the included systematic reviews mentioned the bioavailability of curcumin in their results, and only 6.4% provided quantitative data obtained by subgroup analysis where interventions were divided into two to three subgroups based on curcumin bioavailability. Among those, we found several studies in which the classification of interventions was conducted inaccurately. In the remaining studies, outcomes measured were inflammatory markers, pain, health-related quality of life scores, or anthropometric and cardiometabolic parameters (as detailed in Table 3). This is a very limited set of data, given the large number of studies using curcumin as an intervention for various health conditions. In most of the studies, bioavailability-enhanced formulations were shown to be more effective or the only ones exhibiting significant effects. Such beneficial effects are likely to be lost in studies reporting an overall analysis of curcumin products with different bioavailability profiles.

Our analysis has also revealed that the included systematic reviews were poorly reported. For example, in the methods of 37% of included systematic reviews, participants were not reported, or their description was incomplete. From other parts of the review, such as title and other sections, it could be gathered what kind of participants were included, but it is unfortunate that the authors did not properly report their reviews. Detailed information about the eligible participants should be part of the systematic review methods. Likewise, several systematic reviews have reported results about statistics related to the bioavailability of curcumin, but those statistics were not described in the methods of all those reviews. Future systematic reviews should adhere to the reporting guidance PRISMA 2020 for transparent reporting [24].

Furthermore, descriptions of the eligible interventions in terms of curcumin preparations were very vague in the methods of included reviews. A plethora of solutions have been used to circumvent the curcumin bioavailability issue, such as the addition of metabolism inhibitors, encapsulation in liposomes, micelles, the usage of nanoscale drug delivery systems, etc. However, in many cases, eligible interventions were described with the umbrella terms ‘curcumin’, ‘curcumin or turmeric’, and ‘turmeric’ without precise descriptions. Also, it must be noted that turmeric has a complex chemical composition, with essential oils and curcuminoids being two major groups of compounds that exhibit distinct biological activities [34]. Additional errors were found in that respect in reviews claiming that they studied the effect of curcumin. Some reviews included studies that analyzed curcumin in combination with other co-interventions, such as various mixtures. Some reviews erroneously included studies that analyzed a curcuminoid-free extract of turmeric comprising turmerosaccarides [29,37,38,39].

Our analysis of the mentions of bioavailability in various parts of the analyzed reviews indicated that most reviews reported something about bioavailability in the discussion (Figure 3). Thus, even though most authors are aware of the issues of curcumin bioavailability, they mostly provide general comments about the problems related to this bioavailability. This awareness about the variable bioavailability of curcumin formulations should be translated into the methods and results of systematic reviews, beyond simply commenting on the issue in the discussion.

The limitations of this study include the fact that we only searched PubMed to retrieve systematic reviews on curcumin. As this study was not a systematic review, but a methodological study, we considered it appropriate to search in a single database. It has recently been shown that PubMed could be used as a principal search system [40]. Furthermore, we used inclusion criteria that did not include all curcumin-containing products. This was carried out to find systematic reviews on homogenous curcumin interventions without co-interventions other than methods for enhancing bioavailability. To ensure transparency and detailed insight into our methods, we have enclosed a detailed list of included and excluded studies, with reasons.

## 4. Materials and Methods

### 4.1. Study Design

This was a methodological study in which the unit of analysis was scholarly articles reporting systematic reviews.

### 4.2. Protocol Registration

We published the protocol on Open Science Framework (OSF) Registries (link: https://osf.io/kqxg4; access date: 26 January 2024) before the study commenced.

### 4.3. Inclusion Criteria

We included systematic reviews with or without a meta-analysis where curcumin was used on humans via oral ingestion for systemic absorption and analyzed as an intervention or a comparator, regardless of the types of participants and types of outcomes that were used in a trial. As there is no consensus definition of a systematic review [23], we included all reports that were self-labeled as a systematic review (or a meta-analysis).

### 4.4. Exclusion Criteria

We excluded studies using turmeric rhizome/powders (dietary or supplemented). We excluded studies where curcumin was used as a part of a complex intervention in combination with other substances, other than those added to improve bioavailability. We excluded systematic reviews where curcumin was used via administration modes other than oral ingestion for systemic absorption, such as curcumin taken in the mouth only as a local intervention in the oral cavity or the topical, intravenous, or rectal application of curcumin. We excluded systematic reviews that included exclusively preclinical studies (in silico, in vitro, or animal studies). However, if a systematic review included both human and preclinical/non-human studies, then they were considered eligible. We excluded protocols of systematic reviews if retrieved via our search.

### 4.5. Changes between the Protocol and the Review

Here, we report the changes made to the initial protocol registered on Open Science Framework (OSF) Registries during the preparation of the review. Additional exclusion criteria were applied as follows: we excluded studies using turmeric rhizome (dietary or supplemented), reviews that exclusively analyzed preclinical studies (in silico, in vitro, or animal studies), studies where curcumin was used as a part of complex intervention with other substances, and protocols of systematic reviews.

### 4.6. Search

We searched PubMed on 16 November 2022 by using the following search syntax: curcumin OR curcuma OR turmeric OR theracurmin OR tetrahydrocurcumin OR NCB-02 OR Curcuma domestica Val. OR Curcuma xanthorrhiza OR diferuloylmethane OR curcuminoids OR Biocurcumax OR biocurcumin OR BCM-95 OR BCM-095. We combined it with the filter for systematic reviews and meta-analyses.

### 4.7. Screening

All bibliographic records found with this search were retrieved. Screening for eligible records was conducted in two phases. In the first phase, two authors (V.B.P., E.K.F.) independently screened the titles and abstracts of all records to assess whether they fulfilled the inclusion criteria. For each record, they indicated whether they considered it to be eligible, maybe eligible, or not eligible. We then retrieved full texts of all records assessed as eligible or maybe eligible. In the second screening phase, full texts were screened by two authors (V.B.P., E.K.F.) independently. Disagreements during the screening of full texts were discussed by two authors. Additional authors (I.B., L.P.) were included in the resolution of discrepancies. Reasons for excluding records from the study on the level of full text are recorded in Supplementary File S1.

### 4.8. Data Extraction

For data extraction, we created a data extraction table, which was piloted on a sample of 10 reviews that were included in the study. Two authors tested data extraction (V.B.P., A.J.K.), and another two authors verified the data extraction table (E.K.F., L.P.). The data extraction table was revised iteratively.

For each included study, one author extracted data (V.B.P. and E.K.F. participated in this step) and a second author verified the data extraction (E.K.F., I.B., and A.J.K. participated in this step).

We extracted the following data: the last name of the first author; year of publication; study title; number of studies included that correspond to our inclusion criteria; types of studies included—categorized as randomized controlled trials (RCTs), non-randomized studies (NRSs), or both; number of participants; type of participants included; type of curcumin product that was specified in the review methods as eligible, either as an intervention or a comparator (copied verbatim); whether bioavailability was mentioned in the review methods (yes/no)—if yes, copied verbatim; whether any statistical analyses were mentioned in the methods regarding bioavailability enhancement methods (yes/no)—if yes, copied verbatim; whether bioavailability was mentioned in the review results (yes/no)—if yes, copied verbatim; whether bioavailability was mentioned in the discussion (yes/no)—if yes, copied verbatim; whether bioavailability was mentioned in the conclusion (yes/no)—if yes, copied verbatim; statement of funding for the review (not reported/reported)—if reported, category of funding (commercial, non-commercial, mixed, no funding); conflicts of interest (not reported/reported)—if reported, was at least one author funded/supported in any way by a sponsor of the investigated product (yes/no). After data extraction, we categorized the responses and presented the data narratively. The text was categorized by using the themes that were mentioned in the articles; we did not define any categories prospectively.

### 4.9. Statistics

Data were presented as numbers and frequencies, medians, and ranges. MedCalc Software for Windows (v. 11.5.1.0; MedCalc Software, Broekstraat 52, 9030 Mariakerke, Belgium) was used for analyses.

### 4.10. Raw Data

All raw data collected within the study are presented in Supplementary File S2.

## 5. Conclusions

Despite a relatively large number of systematic reviews investigating oral curcumin interventions, few of them have considered bioavailability as an important factor related to the assessment of efficacy and safety. Based on the wealth of existing data, curcumin appears to be beneficial to human health. However, future systematic reviews should be carefully designed to provide reliable recommendations for its clinical use. Firstly, investigators should be aware of the heterogeneity of curcumin products arising from novel bioavailability-improved curcumin formulations, and they should specify precisely what kinds of interventions are eligible for their study. Bioavailability-improved curcumin formulations should preferably be supported by pharmacokinetic studies so that the dosages can be translated to active compound concentrations in the blood. Secondly, systematic reviews should plan in their methods to conduct relevant subgroup and sensitivity analyses to ensure that significant effects are not lost. The results of the existing systematic reviews on curcumin, in terms of their attention to curcumin bioavailability, should be taken with caution.

## Figures and Tables

**Table 1:** Characteristics of included systematic reviews (N = 171).

Variable		Result
Type of included studies, N (%) *	RCTs	144 (84)
	Both RCTs and NRSs	27 (16)
Number of studies included, median (range)		6 (1 to 29)
Number of participants included, median (range)		380 (8 to 13,594)
Type of curcumin product specified in methods, N (%)	Curcumin	56 (33)
	Not reported	36 (21)
	Curcumin/turmeric	17 (9.9)
	Turmeric	3 (1.8)
	Other	59 (35)
Statement about review funding reported? N (%)	Yes	119 (70)
	No	52 (30)
Category of funding, N (%)	Commercial	1 (0.6)
	Non-commercial	57 (33)
	Mixed	3 (1.8)
	No funding	58 (34)
	Not applicable	52 (30)
A “conflict of interest” statement reported? N (%)	Yes	147 (86)
	No	24 (14)
If “conflict of interest” reported, was at least one author funded/supported in any way by a sponsor of the investigated product? N (%)	Yes	7 (4.1)
	No	140 (82)

* Acronyms: NRS = non-randomized study, RCT = randomized controlled trial.

**Table 2:** Categorization of bioavailability mentioned in included systematic reviews (N = 171).

Study Section Mentioning Bioavailability	Categorization	N (%)
Methods *	Inclusion criteria	6 (3.5)
	Statistics regarding bioavailability	6 (3.5)
	Heterogeneity	2 (1.2)
	Categorization of interventions	1 (1.2)
	Data on bioavailability in included studies not available	1 (0.6)
	Assessing strength of study methodology	1 (0.6)
Results **	Intervention description	19 (11)
	Results of subgroup analysis for bioavailability	10 (5.8)
	Results for studies using bioavailability enhancement	7 (4.1)
	Reporting plasma levels of curcumin	2 (1.2)
	Results of a sensitivity analysis for bioavailability	1 (0.6)
	Methodological assessment	1 (0.6)
	Lack of studies that have evaluated bioavailability	1 (0.6)
	Stratification of results	1 (0.6)
	Explaining low oral availability of curcumin	1 (0.6)
Discussion ***	General comments about curcumin bioavailability	68 (40)
	Description of included interventions	22 (13)
	Results of included studies	19 (11)
	Recommendations for future trials	15 (8.8)
	Heterogeneity of studies in terms of bioavailability	10 (5.8)
	Limitations due to using low bioavailability product	4 (2.3)
	Lack of studies analyzing bioavailability/bioavailability-enhanced formulations	2 (1.7)
	Lack of bioavailability evaluation in included studies	2 (1.7)
Conclusions ****	Summary of results regarding bioavailability	8 (4.7)
	More studies are needed with products with high bioavailability	7 (4.1)
	More bioavailable product forms are needed	5 (2.9)
	Future research needs to compare various formulations regarding bioavailability	4 (2.3)
	Low bioavailability hinders effectiveness of the studied intervention	1 (0.6)
	Future studies need to explore the effect of availability	1 (0.6)
	Curcumin in more bioavailable form can produce effect	1 (0.6)
	Poor availability of curcumin is an issue	1 (0.6)

* While 16 studies mentioned bioavailability, the numbers add up to 17 because 1 study used two of these categories. ** In 41 studies that mentioned bioavailability in results, there were 43 categories for this data item. *** In 97 studies that mentioned bioavailability in discussion, there were 142 categories for this data item. **** In 23 reviews that mentioned bioavailability in conclusions, there were 28 categories for this data item.

**Table 3:** Overview of results of the subgroup analysis performed to assess the effect of bioavailability of curcumin formulation. The number of RCTs using specific curcumin formulation that was included in each subgroup is given in brackets.

Study	Outcomes Measured	Subgroup Analysis				Result
		Subgroup 1		Subgroup 2		
Derosa, 2016 [30]	Interleukin-6 concentration	*Bioavailability-enhanced* curcumin plus piperine (6), Meriva (1)		*Unformulated curcumin* unformulated curcumin (3)		Formulation does not significantly affect the IL-6-lowering effect of curcuminoids
Ferguson, 2021 [26]	Proinflammatory markers	*Bioavailability-enhanced* *CRP:* curcumin plus piperine (5), Longvida (3), Acumin (2), nanomicelle curcumin (2), nanocurcumin (1), BCM-95 curcumin (1), curcumin micelle (1), Meriva (1) *IL-6:* curcumin plus piperine (1), nanocurcumin (1), nanomicelle curcumin (1), curcumin micelles (1), Longvida (1) *TNF-α*: curcumin plus piperine (4), nanomicelle curcumin (2), nanocurcumin (1), Longvida (1) *IL-8:* 3 curcumin plus piperine (1)		*Nonbioavailability-enhanced* *CRP:* unformulated curcumin (4), turmeric (2) *IL-6:* unformulated curcumin (2), turmeric (2) *TNF-α*: unformulated curcumin (2), turmeric (1) *IL-8:* curcumin (1), turmeric (1)		Bioavailability-enhanced formulations result in larger extent of reduction in CRP and comparable reductions in IL-6 and TNF-α; significant reduction in IL-8 was observed only for nonbioavailability enhanced interventions
Sahebkar, 2014b [31]	CRP concentration	*Bioavailability-improved formulations* curcumin plus piperine (2), Longvida (1), Meriva (1)		*Non-improved formulations* unformulated curcumin (2)		Significant CRP lowering effects observed only for bioavailability improved formulations
Sahebkar, 2016a [32]	TNF-α concentration	*Bioavailability-enhanced forumulations* cucumin plus piperine (5)		*Unformulated curcumin* unformulated curcumin (3), turmeric (1)		Larger TNF-α-lowering effect size for bioavailability-improved formulations, but the difference is not of statistical significance
Sahebkar, 2016 [33]	Analgesic effect	*Bioavailability-improved formulations* curcumin plus piperine (2), Meriva (1)		*All products* unformulated curcumin (5), curcumin plus piperine (2), Meriva (1)		Larger effect size for pain reduction for bioavailability improved formulations
Sadeghian, 2021 [27]	HRQOL scores *	*High bioavailability* curcumin plus piperine (3), SinaCurmin (1), BCM-95 (2), CurQfen (2)		*Low bioavailability* unformulated curcumin (4)		Curcumin increased HRQOL for high-bioavailability formulations
		**Subgroup 1**	**Subgroup 2**		**Subgroup 3**	
Sun, 2022 [34]	Anthropometric and cardiometabolic parameters **	*Low-bioavailability* unformulated curcumin (11), turmeric powder (2)	*High-bioavailability* *phospholipid* curcumin/Meriva (5), curcumin plus piperine (4), curcumin micelles (1), Theracurmin (1), BCM-95 (1), curcumin β-cyclodextrin complex (1)		*Nanocurcumin* nanocurcumin (5)	Improvements in BW, BMI, and the levels of FPG, HbA1c, INS, HOMA-IR, HDL-C and Hs-CRP; greater reductions for nanocurcumin group were observed in FPG, INS, TC and LDL-C ***

* HRQOL = health-related quality of life. ** Depending on the data availability for each parameter subgroup including variable number of studies (2–11). *** BW = body weight; BMI = body mass index; FPG = fasting plasma glucose; HbA1c = glycosylated hemoglobin; INS = insulin; HOMA-IR = homeostasis model assessment—insulin resistance; TC = total cholesterol; HDL-C = high-density lipoprotein cholesterol; LDL-C = low-density lipoprotein cholesterol; Hs-CRP = high-sensitivity C-reactive protein.

## Data Availability

All raw data collected and analyzed within the study are available in Supplementary File S2.

## Supplementary Materials

The following supporting information can be downloaded at: https://www.mdpi.com/article/10.3390/ph17020164/s1, Supplementary File S1: List of excluded studies with reasons; Supplementary File S2: Detailed information about included studies, with all raw data extracted during this study.
